# Whole-exome sequencing in a Turkish autism spectrum disorder cohort: a retrospective study of molecular diagnostic yield in isolated and complex presentations

**DOI:** 10.1186/s12888-026-08739-x

**Published:** 2026-10-13

**Authors:** Mehmet Burak Mutlu, Aslıhan Sanrı, Oğuzhan Yaralı, Özlem Sezer, Fethiye Kılıçaslan, Hakan Gümüş, Sefer Kumandaş, Özlem Öz

**Affiliations:** 1 Detagen Genetic Diseases Evaluation Center, Tacettin Veli, Deliklitaş Cd. No: 12/A, Melikgazi, Kayseri 38000 Türkiye; 2 Department of Pediatric Genetics, Samsun Education and Research Hospital, Samsun, Türkiye; 3 Department of Medical Genetics, Erzurum Regional Training and Research Hospital, Erzurum, Türkiye; 4 Department of Medical Genetics, Samsun University Faculty of Medicine, Samsun, Türkiye; 5 Department of Child and Adolescent Psychiatry, Faculty of Medicine, Harran University, Şanlıurfa, Türkiye; 6 Department of Pediatrics, Division of Pediatric Neurology, Faculty of Medicine, Erciyes University, Kayseri, Türkiye; 7 Department of Medical Genetics, Harran University Faculty of Medicine, Şanlıurfa, Türkiye

**Keywords:** Autism spectrum disorder, Whole-exome sequencing, Molecular diagnosis, Neurodevelopmental disorders, Psychiatric molecular genetics

## Abstract

**Background:**

Whole-exome sequencing (WES) can identify molecular etiologies in autism spectrum disorder (ASD), particularly when ASD occurs with developmental delay/intellectual disability (DD/ID), epilepsy, dysmorphism, congenital anomalies, or neuroimaging abnormalities. Data on WES yield and candidate-gene findings in Turkish ASD cohorts remain limited. We assessed molecular diagnostic yield and variant spectrum in a Turkish clinical ASD cohort, with comparison of isolated and complex ASD presentations.

**Methods:**

This retrospective cohort study included 320 individuals with ASD evaluated by WES between 2020 and 2025. Patients were classified as having isolated or complex ASD based on co-occurring neurodevelopmental, neurological, dysmorphic, congenital, or systemic findings. Variants were interpreted using ACMG/AMP criteria. Established molecular diagnoses required pathogenic or likely pathogenic (P/LP) variants associated with OMIM-defined disorders or occurring in genes with definitive, strong, or moderate ClinGen evidence for a neurodevelopmental disorder (NDD). Categorical comparisons used two-sided Fisher’s exact test.

**Results:**

Established molecular diagnoses were identified in 104/320 patients (32.5%; 95% confidence interval [CI], 27.4–37.9%), including 2/22 patients with isolated ASD (9.1%; 95% CI, 1.1–29.2%) and 102/298 patients with complex ASD (34.2%; 95% CI, 28.9–39.9%; *p* = 0.017). An additional 12 patients had P/LP variants in candidate or emerging genes, resulting in an overall P/LP finding rate of 116/320 (36.3%; 95% CI, 31.0–41.8%). Variants of uncertain significance were identified in 147 patients, while no ASD/NDD-related variant was identified in 57 patients.

**Conclusions:**

WES supports etiological clarification in clinically complex ASD. Established molecular diagnoses were substantially more frequent in complex than isolated ASD. Candidate and emerging gene findings should be reported separately and interpreted as hypothesis-generating.

**Supplementary Information:**

The online version contains supplementary material available at https://doi.org/10.1186/s12888-026-08739-x.

## Background

Autism spectrum disorder (ASD) is a highly heritable neurodevelopmental condition characterized by substantial phenotypic heterogeneity, including social communication deficits and restricted, repetitive behaviors [1, 2]. The genetic architecture of ASD and broader neurodevelopmental disorders (NDDs) is highly complex, involving de novo and inherited single-nucleotide variants (SNVs), small insertions/deletions, copy-number variants (CNVs), non-coding variation, and polygenic risk from common variants. Large-scale exome and genome sequencing studies have identified numerous ASD/NDD risk genes and convergent biological pathways, particularly those related to synaptic function, transcriptional regulation, and chromatin remodeling [1–6].

Current genetic evaluation strategies for ASD commonly include *FMR1* CGG repeat testing, chromosomal microarray analysis (CMA), and sequencing-based approaches such as targeted gene panels or whole-exome sequencing (WES) [7–11]. Fragile X syndrome due to *FMR1* CGG repeat expansion accounts for only a small proportion of ASD cases, with reported diagnostic yields generally ranging from approximately 0.5% to 2% in unselected ASD cohorts [12]. Although CMA has traditionally been used as a first-tier test in ASD/NDD workups, sequencing-based approaches, particularly WES, are increasingly used as early-tier or complementary tests because they provide higher diagnostic yields in clinically complex ASD/NDD cohorts [1, 5, 6, 13–15]. Meta-analytic studies and clinic-based comparisons have shown that sequencing approaches, including multigene panels and WES, generally outperform CMA in diagnostic yield, especially in syndromic ASD and developmental delay/intellectual disability (DD/ID) cohorts [5, 15, 16]. Direct comparative data from ASD cohorts also support the diagnostic advantage of WES over CMA in selected clinical settings [13].

The diagnostic yield of WES in ASD varies substantially according to study design, sequencing strategy, and phenotypic complexity. Across ASD and broader NDD populations, exome-based diagnostic yields have been reported from the mid-teens to the upper 30% range, with higher yields observed in individuals with DD/ID, epilepsy, dysmorphic features, congenital anomalies, abnormal neuroimaging, or other neurological and systemic findings [1, 14–16]. Trio-based WES may further increase diagnostic yield by facilitating the interpretation of de novo variants, clarifying inheritance patterns, and improving the prioritization of clinically relevant variants, including CNVs [14, 17]. Therefore, distinguishing isolated ASD from complex or syndromic ASD is important for interpreting WES results and for avoiding over- or underestimation of diagnostic yield in heterogeneous ASD cohorts [1, 15–17].

Beyond establishing molecular diagnoses in known OMIM- or ClinGen-defined disorders, WES can also identify rare variants in candidate or emerging ASD/NDD genes. Such findings may contribute to gene discovery, support the refinement of genotype–phenotype correlations, and expand the clinical spectrum of rare NDDs. Regional and population-based studies from India and Latin America have highlighted the value of implementing WES in ASD/NDD cohorts, particularly in clinically complex cases [13, 18, 19]. However, data on WES-based diagnostic yield and candidate gene spectrum in ASD cohorts from Türkiye and neighboring regions remain limited, despite the potential contribution of population-specific variants, recessive inheritance, and complex clinical presentations.

In this study, we evaluated the diagnostic yield and molecular spectrum of WES in a Turkish ASD cohort, stratified according to isolated and complex/syndromic ASD presentations. We further assessed OMIM- and ClinGen-defined diagnostic findings, candidate ASD/NDD-associated genes, rare or apparently novel variants, and findings contributing to genotype–phenotype correlation.

## Methods

### Patients

This retrospective cohort study was conducted at Harran University Faculty of Medicine Hospital and Detagen Genetic Diseases Evaluation Center. The study protocol was approved by the Harran University Clinical Research Ethics Committee (HRÜ:23/02/13), and the study was conducted in accordance with the principles of the Declaration of Helsinki. Written informed consent for genetic testing and the use of clinical data for research purposes was obtained from all participants or from their parents/legal guardians.

Patients were recruited from the child and adolescent psychiatry and clinical genetics departments. The study population comprised individuals diagnosed with ASD who underwent WES over a five-year period between 2020 and 2025. The diagnosis of ASD was established by child and adolescent psychiatry specialists according to the Diagnostic and Statistical Manual of Mental Disorders, Fifth Edition (DSM-5) criteria.

Clinical data were retrospectively reviewed from medical records, including age, sex, family history, parental consanguinity, developmental history, DD/ID, epilepsy, dysmorphic features, congenital anomalies, neurological findings, neuroimaging abnormalities, and other systemic manifestations, when available. Classification as isolated ASD required sufficient clinical documentation to assess developmental status, epilepsy, dysmorphic and congenital features, neurological findings, and other relevant systemic manifestations. Only individuals for whom the available clinical records were sufficient to exclude major accompanying neurodevelopmental, neurological, dysmorphic, congenital, or systemic findings were classified as having isolated ASD. Patients were classified as having complex or syndromic ASD when ASD co-occurred with features such as DD/ID, epilepsy, hypotonia or other motor abnormalities, dysmorphic features, congenital anomalies, abnormal neuroimaging, microcephaly/macrocephaly, or multisystem involvement.

### Whole-exome sequencing and variant interpretation

Genomic DNA was extracted from peripheral blood samples using the QIAamp DNA Mini Kit (Cat. No. 51304; Qiagen, Hilden, Germany), according to the manufacturer’s instructions. DNA concentration was quantified using the Qubit dsDNA HS Assay Kit. The total DNA input for library preparation was 25 ng for the Twist workflow and 50 ng for the Vazyme and Genetopia workflows. Exome libraries were prepared using Twist, Vazyme, or Genetopia library preparation workflows. Different validated library preparation and sequencing workflows were used over the five-year study period according to routine laboratory workflow, instrument and reagent availability, and procurement arrangements. Sequencing was performed in paired-end mode with 2 × 150 bp reads on Illumina NovaSeq and GeneMind SurfSeq platforms, achieving an average sequencing depth of approximately 70×–100×.

Raw sequencing data were processed and aligned to the GRCh38/hg38 human reference genome. Variant analysis was focused on protein-coding exons and adjacent intronic regions extending ± 10 bp from exon–intron boundaries. Raw sequencing reads were aligned to the GRCh38/hg38 reference genome using the Burrows–Wheeler Aligner (BWA), and SNVs and small insertions/deletions were called using the Genome Analysis Toolkit (GATK). Called variants were subsequently annotated using Ensembl Variant Effect Predictor (VEP). CNVs were evaluated using GATK and ExomeDepth, when applicable, and clinically relevant CNVs identified by WES-based CNV analysis were confirmed by chromosomal microarray analysis (CMA).

Following primary filtering, candidate variants were evaluated using publicly available and clinically curated databases, including ClinVar, the Database of Genomic Variants, DECIPHER, VarSome, Franklin, and gnomAD. Missense variants were further assessed using multiple in silico prediction tools, including SIFT, PolyPhen-2, MutationTaster, and Combined Annotation Dependent Depletion (CADD) scores. Potential effects on splicing were evaluated using SpliceAI. Rare variants were prioritized using a minor allele frequency (MAF) threshold of < 0.1% for dominant inheritance models. For recessive inheritance models, population frequency was evaluated together with genotype-specific observations in gnomAD. In autosomal recessive disorders, particular attention was given to homozygous variants and their homozygous counts, whereas in X-linked recessive disorders, homozygous and hemizygous variants were evaluated together with their genotype-specific observations in gnomAD. A single fixed MAF threshold was not applied to recessive models. Variant interpretation was performed in the context of the patient’s clinical phenotype, inheritance pattern, variant type, population frequency, predicted functional effect, and available family segregation data. Sequence variants were classified primarily according to the ACMG/AMP guidelines, with ClinGen Sequence Variant Interpretation (SVI) recommendations consulted when applicable for the application and strength assignment of specific evidence criteria [20]. Clinically relevant CNVs were interpreted according to ACMG/ClinGen technical standards for constitutional copy number variant interpretation, when applicable. Genotype–phenotype correlations and candidate gene assessments were performed using OMIM, ClinGen, and SFARI Gene databases. Gene–disease validity was assessed using the ClinGen gene–disease validity classifications available at the time of final analysis, with particular consideration of relationships classified as Definitive, Strong, or Moderate. We did not independently perform gene–disease validity curation using the ClinGen General SOP, and specific ClinGen Expert Panel assessments were not systematically used as a separate criterion.

All patients underwent proband-only WES; no trio- or duo-based WES was performed. Parental samples were therefore not sequenced concurrently with the proband. Instead, targeted parental segregation analysis was performed post hoc, when indicated, to determine inheritance, clarify variant phase, and confirm suspected compound heterozygosity.

In our laboratory, clinical reanalysis is routinely considered after at least one year from the initial interpretation, or earlier when relevant new clinical or genomic evidence becomes available. This one-year interval reflects routine institutional laboratory practice and was not selected specifically for the present study. Reanalysis events were not systematically logged, and a uniform cohort-wide reanalysis immediately before manuscript submission was not performed. Candidate/emerging genes were considered collectively rather than as formally distinct categories and were defined as genes lacking an OMIM-defined disorder and lacking definitive, strong, or moderate ClinGen gene–disease validity evidence for NDDs at the time of final analysis. Variants in these genes were classified at the variant level according to ACMG/AMP criteria; however, they were not considered established molecular diagnoses. Categorical variables were compared using two-sided Fisher’s exact test, and a *p* value of < 0.05 was considered statistically significant. Exact 95% confidence intervals (CIs) for proportions were calculated using the Clopper–Pearson method.

## Results

A total of 320 patients met the inclusion criteria, including 196 males (61.3%) and 124 females (38.8%). Of the 320 patients, 22 were classified as having isolated ASD, whereas 298 were classified as having complex ASD. Sufficient phenotypic documentation to apply the predefined isolated-ASD classification criteria was available for all 22 individuals in the isolated-ASD subgroup. Parental consanguinity was documented in 33 of 320 patients (10.3%). Among these individuals, homozygous variants were identified in 23 patients and compound-heterozygous variants in two patients, whereas no ASD/NDD-related variant was identified in the remaining eight individuals. Post hoc parental segregation analysis was performed in 36 of 320 patients (11.3%), including 2 of 22 patients with isolated ASD (9.1%) and 34 of 298 patients with complex ASD (11.4%). The availability of parental segregation data was therefore similar between the isolated- and complex-ASD subgroups. Among the 320 patients, 116 (36.3%) harbored a pathogenic or likely pathogenic (P/LP) variant, 147 (45.9%) harbored only a variant of uncertain significance (VUS) in a gene potentially related to ASD/NDD, and no ASD/NDD-related variant was identified in the remaining 57 patients (17.8%). The reported VUS category reflects the classifications used in the final dataset analyzed for this study. However, the number of cases that underwent prior clinical reanalysis and the number of individual upgrades or downgrades could not be reliably reconstructed because reanalysis events were not systematically recorded. These categories were mutually exclusive, and each patient was assigned to a single final molecular finding category.

Established molecular diagnoses were identified in 104 of 320 patients (32.5%; 95% CI, 27.4–37.9%) based on pathogenic or likely pathogenic variants in OMIM-defined disorders or genes with definitive, strong, or moderate ClinGen evidence for NDDs. Among patients with isolated ASD, established molecular diagnoses were identified in 2 of 22 patients (9.1%; 95% CI, 1.1–29.2%), compared with 102 of 298 patients with complex ASD (34.2%; 95% CI, 28.9–39.9%; two-sided Fisher’s exact test, *p* = 0.017).

**Fig. 1:**
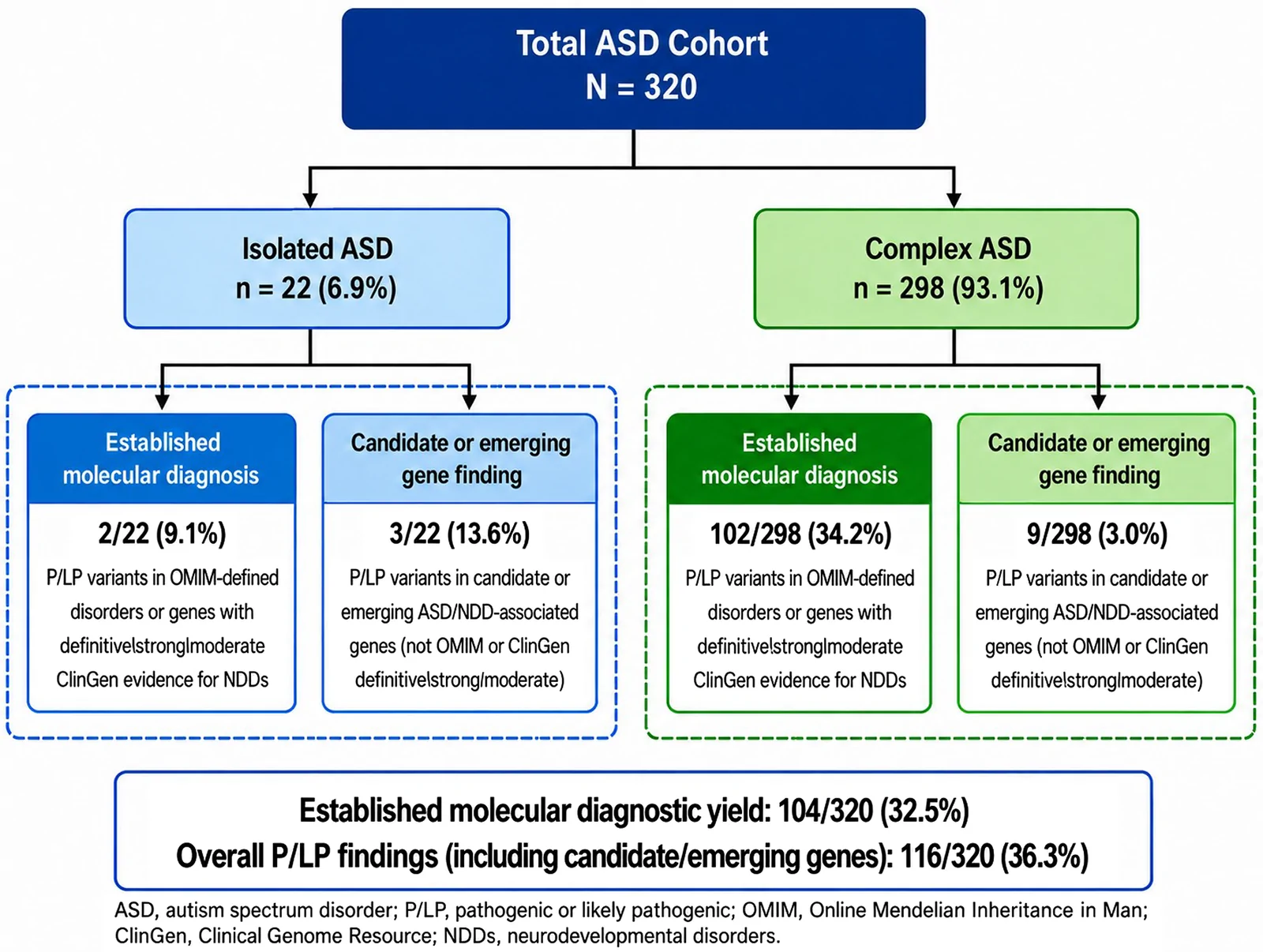
Cohort overview and molecular findings. Flowchart of the 320 individuals with ASD included in the study. Established molecular diagnoses, defined by P/LP variants in OMIM-defined disorders or genes with definitive, strong, or moderate ClinGen evidence for NDDs, were identified in 104 patients (32.5%; 95% CI, 27.4–37.9%). Candidate/emerging gene findings were identified in 12 patients (3.8%), including 3 of 22 individuals with isolated ASD (13.6%) and 9 of 298 individuals with complex ASD (3.0%). Established molecular diagnoses were identified in 2 of 22 individuals with isolated ASD (9.1%; 95% CI, 1.1–29.2%) and 102 of 298 individuals with complex ASD (34.2%; 95% CI, 28.9–39.9%). When established and candidate/emerging findings were considered together, potentially relevant findings were observed in 5 of 22 individuals with isolated ASD (22.7%; 95% CI, 7.8–45.4%) and 111 of 298 individuals with complex ASD (37.2%; 95% CI, 31.7–43.0%). Overall, P/LP findings, including candidate/emerging gene findings, were identified in 116 of 320 patients (36.3%; 95% CI, 31.0–41.8%)

In addition, P/LP variants were identified in candidate or emerging ASD/NDD-associated genes in 12 patients, including 3 patients with isolated ASD and 9 patients with complex ASD. When established and candidate findings were considered together, potentially relevant molecular findings were observed in 5 of 22 patients with isolated ASD (22.7%; 95% CI, 7.8–45.4%) and 111 of 298 patients with complex ASD (37.2%; 95% CI, 31.7–43.0%), for a total of 116 of 320 patients (36.3%; 95% CI, 31.0–41.8%). The difference between isolated and complex ASD was not statistically significant when established and candidate findings were considered together (22.7% vs. 37.2%; two-sided Fisher’s exact test, *p* = 0.250). The cohort overview and molecular findings are shown in Fig. 1.

A total of 56 variants were classified as apparently novel, as they were absent from PubMed, ClinVar, and gnomAD and lacked rs identifiers at the time of analysis. These variants are presented in Table 1.

**Table 1:** Apparently novel pathogenic and likely pathogenic sequence variants identified in the ASD-WES cohort

Gene	Transcript	cDNA Change	Protein Change	Zygosity	ACMG/AMP Classification
ARHGEF9	NM_001353921.2	c.1001G>A	p.Trp334Ter	Hemizygous	LP
AUTS2	NM_015570.4	c.1211T>G	p.Leu404Ter	Heterozygous	LP
AUTS2	NM_015570.4	c.1289del	p.Pro430ArgfsTer43	Heterozygous	LP
CACNA2D2	NM_006030.4	c.2402-1G>C*	—	Heterozygous	LP
CHAMP1	NM_032436.4	c.1545G>A	p.Trp515Ter	Heterozygous	LP
CHD8	NM_001170629.2	c.6880del	p.Val2294Ter	Heterozygous	LP
CHD8	NM_001170629.2	c.2142+2T>C	—	Heterozygous	LP
CNOT3	NM_014516.4	c.1117C>T	p.Gln373Ter	Heterozygous	P
CNTN6	NM_001289080.2	c.188dup	p.Gln64AlafsTer7	Heterozygous	LP
CNTNAP2	NM_014141.6	c.2371del	p.Cys791AlafsTer40	Heterozygous	LP
CTNNB1	NM_001904.4	c.357del	p.Thr120LeufsTer13	Heterozygous	LP
DDX3X	NM_001356.5	c.1729G>T	p.Glu577Ter	Heterozygous	LP
DIP2A	NM_015151.4	c.3798+1del	—	Heterozygous	LP
DTNA	NM_001386795.1	c.1714_1717del	p.Gln572TrpfsTer5	Heterozygous	LP
DYRK1A	NM_001347721.2	c.887_888insGCAA	p.Ile296MetfsTer4	Heterozygous	LP
EIF5A	NM_001970.5	c.381C>A	p.Tyr127Ter	Heterozygous	LP
EXOC8	NM_175876.5	c.1654C>T	p.Gln552Ter	Homozygous	LP
FBXW7	NM_018315.5	c.52_56del	p.Gly18PhefsTer8	Heterozygous	LP
FOXG1	NM_005249.5	c.643T>G	p.Phe215Val	Heterozygous	LP
FOXG1	NM_005249.5	c.570_572del	p.Ile190del	Heterozygous	LP
GATAD2B	NM_020699.4	c.731del	p.Gly244ValfsTer17	Heterozygous	LP
GIGYF1	NM_001375765.1	c.2140C>T	p.Gln714Ter	Heterozygous	LP
GRID2IP	NM_001145118.2	c.1588del	p.Leu530Ter	Heterozygous	LP
HNRNPR	NM_005826.5	c.1642_1643dup	p.Pro549ValfsTer22	Heterozygous	LP
IQSEC2	NM_001111125.3	c.2563del	p.Arg855AspfsTer52	Heterozygous	LP
KDM5C	NM_004187.5	c.1726del	p.Leu576SerfsTer83	Hemizygous	LP
KMT2C	NM_170606.3	c.8389_8390del	p.Lys2797GlyfsTer11	Heterozygous	LP
LINS1	NM_001040616.3	c.1870del	p.Gln624LysfsTer4	Homozygous	LP
MECP2	NM_001110792.2	c.622_623insT	p.Thr208IlefsTer40	Heterozygous	LP
MECP2	NM_001110792.2	c.1079_1105delinsC	p.Glu360AlafsTer36	Heterozygous	P
MED13	NM_005121.3	c.5256+2T>C	—	Heterozygous	LP
MED13L	NM_015335.5	c.480_483dup	p.His162Ter	Heterozygous	LP
NAA15	NM_057175.5	c.774G>A	p.Trp258Ter	Heterozygous	P
NBEA	NM_001385012.1	c.29_41del	p.Pro10LeufsTer32	Heterozygous	LP
PATJ	NM_001350145.3	c.5246_5247insTG	p.Tyr1751ProfsTer10	Heterozygous	LP
PCDHAC1	NM_018898.5	c.1622_1623del	p.Ile541LysfsTer7	Heterozygous	LP
PPP3CA	NM_000944.5	c.1242-6_1245dup	p.Glu416PhefsTer6	Heterozygous	LP
PRMT7	NM_019023.5	c.1192-2A>G	—	Homozygous	LP
RBL2	NM_001323611.1	c.18+1G>T	—	Homozygous	LP
RFX3	NM_001282116.2	c.1467_1470del	p.Ser490ProfsTer62	Heterozygous	LP
SATB2	NM_001172509.2	c.474_475del	p.Ser158ArgfsTer50	Heterozygous	LP
SCN8A	NM_001330260.2	c.3998T>C	p.Val1333Ala	Heterozygous	LP
SLC9A9	NM_173653.4	c.1000+2T>A	—	Heterozygous	LP
SRCAP	NM_006662.3	c.7745dup	p.Lys2583Ter	Heterozygous	LP
SRRM2	NM_016333.4	c.1032+1G>A	—	Heterozygous	LP
SYNGAP1	NM_006772.3	c.1010del	p.Lys337ArgfsTer10	Heterozygous	P
TBL1XR1	NM_024665.7	c.570C>G	p.Asp190Glu	Heterozygous	LP
TCF20	NM_001378418.1	c.5723dup	p.Tyr1908Ter	Heterozygous	LP
TCF20	NM_005650.4	c.3662_3663dup	p.Gly1222MetfsTer3	Heterozygous	LP
TCF20	NM_001378418.1	c.4124_4125dup	p.Gly1376LeufsTer14	Heterozygous	LP
TMEM222	NM_032125.3	c.476del	p.Asn159ThrfsTer8	Homozygous	LP
TRIP12	NM_001348323.3	c.3307+2T>A	—	Heterozygous	LP
TRRAP	NM_003496.3	c.5764C>T	p.Gln1922Ter	Heterozygous	LP
UBE3A	NM_130839.5	c.2050_2051del	p.Leu684PhefsTer3	Heterozygous	LP
USP7	NM_003470.3	c.406del	p.Ala136GlnfsTer3	Heterozygous	LP
VPS13B	NM_152564.5	c.10789G>T*	p.Gly3597Ter	Compound Heterozygous	LP

Note. Variants were classified as apparently novel because they were absent from PubMed, ClinVar, and gnomAD and lacked rs identifiers at the time of analysis. P, pathogenic; LP, likely pathogenic; ASD, autism spectrum disorder; WES, whole-exome sequencing. — indicates that a specific protein-level consequence was not assigned for the corresponding splice-site variant. In the *CACNA2D2* case, the apparently novel LP c.2402-1G>C variant was identified together with the previously reported c.244G>A (p.Asp82Asn) variant, classified as a VUS. Parental segregation analysis was not performed; therefore, the phase of these variants could not be confirmed. In the *VPS13B* case, the apparently novel LP c.10789G>T (p.Gly3597Ter) variant was identified together with the previously reported LP c.5011C>T (p.Arg1671Ter) variant, and segregation analysis confirmed that the two variants were in trans. Because Table 1 is restricted to apparently novel P/LP variants, the non-novel partner alleles are not listed as separate entries. Some variants listed in this table occur in candidate or emerging genes and are discussed separately in Table 2; their pathogenicity classifications reflect variant-level ACMG/AMP assessment and do not establish a confirmed molecular diagnosis. An asterisk denotes the apparently novel allele in a case with an additional non-novel partner allele

A comprehensive list of all P/LP sequence variants, VUS, and cases with multiple molecular findings, including compound heterozygous variants, available segregation analyses, and inheritance information, is provided in Additional file 1.

To the best of our knowledge, the *SLC9A6* c.1661+1G>A, *CTCF* c.1103G>A, *OTUD6B* c.379_383del, and *SNAP25* c.596C>G variants have only rarely been reported previously in individuals or families with NDDs. The *CTCF* and *SNAP25* findings represent independent observations with concordant NDD/ASD phenotypes, whereas the *SLC9A6* and *OTUD6B* findings provide additional genotype–phenotype information because ASD was not reported in the previously described individuals or family [21–24].

Clinically relevant CNVs were identified in two patients. One patient harbored a duplication at chromosome 16p13.11, whereas another harbored a deletion at chromosome 22q13.33. Both CNVs were confirmed by CMA. The patients were diagnosed with 16p13.11 microduplication syndrome and Phelan-McDermid syndrome, respectively.

## Discussion

In this Turkish ASD cohort, WES identified established molecular diagnoses in 104 of 320 patients (32.5%). The confirmed diagnostic yield was significantly higher among individuals with complex ASD than among those with isolated ASD (34.2% vs. 9.1%; Fisher’s exact test, *p* = 0.017). This finding is consistent with previous studies showing that DD/ID, epilepsy, dysmorphic features, congenital anomalies, abnormal neuroimaging, and other neurological or systemic manifestations increase the likelihood of identifying an underlying monogenic NDD [1, 5, 14–16].

An additional 12 patients harbored P/LP variants in candidate or emerging ASD/NDD-associated genes. These findings were analyzed separately because the underlying gene–disease relationships remain incomplete or evolving. When candidate findings were included, potentially relevant molecular findings were observed in 22.7% of isolated ASD cases and 37.2% of complex ASD cases; however, this broader comparison was not statistically significant and should not be interpreted as a confirmed diagnostic-yield difference.

Our findings are consistent with previous studies showing that exome sequencing is particularly informative in individuals with ASD accompanied by DD/ID, epilepsy, dysmorphic features, congenital anomalies, or abnormal neuroimaging. However, because this study did not include a direct head-to-head comparison between WES and CMA, it does not demonstrate the superiority of WES over CMA. Rather, the present findings support early use of WES with validated CNV analysis as part of a phenotype-guided diagnostic pathway, while CMA, *FMR1* testing, and other targeted investigations remain complementary when clinically indicated [1, 5–12, 25].

After review of the available published literature, four variants identified in our cohort had only rarely been reported previously in individuals or families with NDDs: *SLC9A6* (NM_001379110.1): c.1661+1G>A, *CTCF* (NM_006565.4): c.1103G>A, *OTUD6B* (NM_016023.5): c.379_383del, and *SNAP25* (NM_130811.4): c.596C>G. These observations may provide additional genotype–phenotype information, particularly when ASD was absent or incompletely characterized in the previously reported individuals. The *SLC9A6* c.1661+1G>A variant was previously reported in a heterozygous female with NDD; however, ASD was not described in that individual [21]. The *CTCF* c.1103G>A variant was reported in a single individual with NDD and ASD, consistent with the phenotype observed in our patient [22]. The *OTUD6B* c.379_383del variant was previously described in homozygous siblings with NDD, although ASD was not reported in that family [23]. Similarly, *SNAP25* c.596C>G was reported in a previous individual with NDD and ASD, consistent with the phenotype of our patient [24]. In contrast, the individual carrying the *NCKAP1* (NM_013436.5): c.2021+1G>A variant had already been reported by Gündoğdu Öğütlü et al. and was included in the present study as part of the overall ASD cohort analysis; therefore, this case does not represent an independent replication [26].

Rare predicted loss-of-function variants were identified in eight candidate/emerging genes with variable levels of evidence for ASD/NDD association. Because variant-level classification cannot be interpreted independently of the strength of the underlying gene–disease relationship, these findings were analyzed separately from established molecular diagnoses and should be regarded as hypothesis-generating pending further segregation, functional, and replication evidence.

Among the candidate or emerging findings, *ABCA13*,* PATJ*,* DIP2A*,* PCDHAC1*,* CNTN6*,* GRID2IP*,* TMPRSS9*, and *XPO1* have varying levels of association-based, functional, animal-model, or limited human genetic evidence relevant to ASD or neurodevelopment [27–45]. However, the currently available evidence is insufficient to establish these genes as definitive monogenic causes of ASD/NDD in the present cohort. Therefore, the identified predicted loss-of-function variants should be interpreted as biologically plausible candidate findings rather than confirmed molecular diagnoses. *CNTN6* requires particular caution because its gene–disease relationship remains disputed, and isolated *CNTN6* variants may act as modifiers rather than fully penetrant disease-causing variants [46–48].

*XPO1* was also discussed descriptively as an emerging rather than established NDD gene in the present study because it does not yet have an OMIM disease designation or ClinGen gene–disease validity curation. Nevertheless, a recent study of 22 individuals with de novo *XPO1* variants, supported by functional data, provides emerging evidence for a dominant NDD characterized by DD/ID, microcephaly, structural brain abnormalities, behavioral problems, and variably ASD or seizures [44, 45]. Therefore, the *XPO1* p.Trp235Ter finding should be regarded as an emerging-gene observation that requires further replication and clinical curation before being considered a confirmed molecular diagnosis.

Additionally, variants were identified in *UNC79*,* MYCBP2*,* UBR5*,* DUSP15*, and *PER1*. None of these genes met our predefined criteria for an established molecular diagnosis, as they were not associated with an OMIM-defined syndrome and did not have definitive, strong, or moderate ClinGen gene–disease validity evidence for NDDs at the time of analysis. Although previous studies have suggested potential associations of *UNC79*, *MYCBP2*, and *UBR5* with neurodevelopmental phenotypes [49–51] and association-based evidence has been reported for *DUSP15* and *PER1* [52, 53], the specific variants identified in our cohort were classified as variants of uncertain significance. Accordingly, these findings were considered candidate-gene observations and were not included in the established molecular diagnostic yield or among the P/LP candidate-gene findings. All candidate genes and variants are presented in Table 2.

**Table 2:** Candidate and emerging gene findings identified in the ASD-WES cohort, including P/LP variants and VUS

Candidate/emerging genes with pathogenic or likely pathogenic variants					
Gene	Transcript	cDNA Change	Protein Change	Zygosity	ACMG/AMP Classification
ABCA13	NM_152701.5	c.9860-2A>G	—	Heterozygous	LP
ABCA13	NM_152701.5	c.9676C>T	p.Gln3226Ter	Heterozygous	LP
ABCA13	NM_152701.5	c.2570T>A	p.Leu857Ter	Heterozygous	LP
ABCA13	NM_152701.5	c.9387del	p.Leu3130Ter	Heterozygous	LP
ABCA13	NM_152701.5	c.1866-2A>G	—	Heterozygous	LP
CNTN6	NM_001289080.2	c.188dup	p.Gln64AlafsTer7	Heterozygous	LP
DIP2A	NM_015151.4	c.3798+1del	—	Heterozygous	LP
GRID2IP	NM_001145118.2	c.1588del	p.Leu530Ter	Heterozygous	LP
PATJ	NM_001350145.3	c.5246_5247insTG	p.Tyr1751ProfsTer10	Heterozygous	LP
PCDHAC1	NM_018898.5	c.1622_1623del	p.Ile541LysfsTer7	Heterozygous	LP
TMPRSS9	NM_001395513.1	c.2331dup	p.Pro778SerfsTer54	Heterozygous	LP
XPO1	NM_003400.4	c.705G>A	p.Trp235Ter	Heterozygous	LP

**Candidate/emerging genes with variants of uncertain significance**					
**Gene**	**Transcript**	**cDNA Change**	**Protein Change**	**Zygosity**	**ACMG/AMP** **Classification**
UNC79	NM_001346218.2	c.4809_4811delinsCAG	p.Thr1604Arg	Heterozygous	VUS
MYCBP2	NM_015057.5	c.1029T>A	p.Asn343Lys	Heterozygous	VUS
UBR5	NM_015902.6	c.1097A>C	p.Lys366Thr	Heterozygous	VUS
DUSP15	NM_080611.5	c.224del	p.His75ProfsTer25	Heterozygous	VUS
PER1	NM_002616.3	c.1681C>T	p.Gln561Ter	Heterozygous	VUS

Note. Pathogenicity classifications refer to variant-level ACMG/AMP assessment. Because the listed genes do not meet the predefined criteria for an established OMIM- or ClinGen-supported NDD, these findings were interpreted as candidate or emerging gene observations rather than confirmed molecular diagnoses

Additionally, our cohort included variants in *DTNA*,* FBXW7*,* EXOC8*, and *PRMT7*, which are associated with defined NDD phenotypes; however, their direct relationship with ASD remains unclear or insufficiently established. The *DTNA* (NM_001386795.1): c.1714_1717del variant identified in our cohort met our predefined criteria for apparent novelty, being absent from PubMed, ClinVar, and gnomAD and lacking an rs identifier at the time of final analysis. No additional pathogenic or likely pathogenic variant in an established ASD/NDD-associated gene was identified in this individual. *DTNA* has been associated with myopathy with myalgia, increased serum creatine kinase, and with or without episodic rhabdomyolysis 2 (OMIM**#**620971). However, monoallelic *DTNA* variants have recently been associated with a mild dominantly inherited muscular dystrophy, with ASD and learning disabilities reported in some individuals carrying the recurrent *DTNA* c.1567_1587del variant [54]. Therefore, the *DTNA* finding in our patient should be interpreted cautiously as a possible contributor to a neuromuscular-neurodevelopmental phenotype rather than as a definitive ASD-causing variant. Although ASD and learning difficulties have been reported in a limited number of individuals with *DTNA*-related disease, additional unrelated cases and functional evidence are required before a specific role in ASD can be established. The present case adds to the limited clinical observations but does not establish a specific role for *DTNA* in ASD.

The monoallelic *FBXW7* variants have recently been associated with a neurodevelopmental syndrome characterized by DD/ID, hypotonia, impaired language, gastrointestinal problems, and variable additional features. Although ASD was diagnosed in one case in the literature, autistic or stereotypic behaviors do not appear to be among the most common or defining manifestations of *FBXW7*-related NDD [55, 56]. The biallelic pathogenic variants in *PRMT7* cause SBIDDS syndrome (Short Stature, Brachydactyly, Intellectual Developmental Disability, and Seizures), an autosomal recessive syndromic NDD characterized by short stature, brachydactyly, DD/ID, hypotonia, seizures, and dysmorphic features. Although ASD has not been established as a recurrent or defining feature of *PRMT7*-related syndrome, experimental data from *Prmt7* knockout mice showing hyperactivity and impaired social interaction provide biological plausibility for a potential contribution to neurodevelopmental and social-behavioral phenotypes [57]. *EXOC8* has been associated with an emerging autosomal recessive NDD characterized mainly by DD/ID, seizures, microcephaly, and cerebral atrophy. However, ASD has not been clearly reported as a recurrent or defining feature in the published *EXOC8*-related cases [58].

Several limitations should be acknowledged. First, the retrospective design and recruitment from tertiary child psychiatry and clinical genetics services likely enriched the cohort for complex ASD and may have increased the observed diagnostic yield. Second, the isolated-ASD subgroup was small, limiting the statistical power of subgroup comparisons. Third, different library preparation kits and sequencing platforms were used during the five-year study period, and no formal cross-workflow comparison of target capture efficiency or coverage bias was performed; therefore, subtle workflow-related differences in variant detection cannot be excluded. In addition, reanalysis was performed according to routine clinical laboratory practice rather than as a standardized study procedure, and the entire cohort did not undergo uniform reanalysis at a single time point immediately before manuscript submission. WES also has limited sensitivity for repeat expansions, balanced structural variants, low-level mosaicism, deep intronic variants, and non-coding regulatory changes. Finally, candidate and emerging gene findings were not uniformly supported by segregation or functional studies and should be interpreted as hypothesis-generating.

## Conclusions

In this Turkish clinical ASD cohort, WES identified established molecular diagnoses in 104 of 320 individuals (32.5%). Established molecular diagnoses were significantly more frequent in complex ASD than in isolated ASD, whereas the difference in potentially relevant findings after inclusion of candidate genes was not statistically significant. These results support early use of WES, preferably with validated CNV analysis, in individuals with ASD accompanied by DD/ID, epilepsy, dysmorphic features, congenital anomalies, or abnormal neuroimaging. Candidate and emerging gene findings should be reported separately from confirmed diagnoses and require further segregation, functional, and replication evidence. CMA, *FMR1* testing, and phenotype-directed targeted tests remain complementary components of a comprehensive ASD genetic evaluation.

## Supplementary Information

Below is the link to the electronic supplementary material.

Supplementary Material 1: Additional file 1: Comprehensive list of pathogenic and likely pathogenic sequence variants, variants of uncertain significance, and cases with multiple molecular findings, including compound heterozygous variants, available segregation analyses, and inheritance information.

## Data Availability

The datasets generated and/or analysed during the current study are not publicly available because they contain potentially identifiable human genomic and clinical data from pediatric participants, and public deposition was not covered by the original informed consent and ethics approval. De-identified summary-level variant data supporting the findings of this study are included in this article and its supplementary information files. Additional de-identified data may be made available from the corresponding author upon reasonable request, subject to approval by the relevant ethics committee and institutional data-sharing regulations.

## Abbreviations

ACMG/AMP: American College of Medical Genetics and Genomics/Association for Molecular Pathology
ASD: Autism Spectrum Disorder
BWA: Burrows–Wheeler Aligner
CI: Confidence Interval
CMA: Chromosomal Microarray Analysis
CNV: Copy-Number Variant
DD/ID: Developmental Delay/Intellectual Disability
GATK: Genome Analysis Toolkit
LP: Likely Pathogenic
MAF: Minor Allele Frequency
NDD: Neurodevelopmental Disorder
OMIM: Online Mendelian Inheritance in Man
P: Pathogenic
P/LP: Pathogenic or Likely Pathogenic
SNV: Single-Nucleotide Variant
SVI: Sequence Variant Interpretation
VEP: Variant Effect Predictor
VUS: Variant of Uncertain Significance
WES: Whole-Exome Sequencing
